# Perovskite Solar Modules for the Residential Sector

**DOI:** 10.1021/acsenergylett.3c02111

**Published:** 2023-10-25

**Authors:** Lucie McGovern, Esther Alarcón-Lladó, Erik C. Garnett, Bruno Ehrler, Bob van der Zwaan

**Affiliations:** † Faculty of Science (HIMS, IOP and/or IAS), University of Amsterdam, Amsterdam 1098 XH, The Netherlands; ‡ Center for Nanophotonics, AMOLF, Amsterdam 1098 XG, The Netherlands; § TNO Energy Transition Studies, Amsterdam 1043 NT, The Netherlands; ∥ School of Advanced International Studies (SAIS), Johns Hopkins University, Bologna 40126, Italy

Perovskite solar cells have received tremendous attention within the solar research field in the past decade, due to their outstanding optoelectronic qualities^1,2^ as well as the exciting prospect of low-cost processing, for instance, with roll-to-roll manufacturing.^3^ After an astonishing first decade of development within the laboratory environment (from technology readiness level 1 to 4), now comes the time for the possible second phase of perovskite photovoltaics (PV), which will ultimately determine whether these model material candidates make their full transformation toward commercial modules. As the interest in perovskite PV expands toward new actors such as industrial companies,^4−6^ policy-makers,^7,8^ and news outlets,^9,10^ the question still remains where exactly these new modules could benefit the solar industry most. With crystalline silicon (c-Si) PV already present on a very large scale at the utility level, we and others have shown that perovskite modules currently offer a relatively small window of opportunity for competition against this incumbent technology,^11,12^ at least within the utility application scale and at the time of this writing. The picture is different when it comes to rapidly growing applications such as building-integrated photovoltaics (BIPV)^13^ and for market segments where silicon PV remains more expensive, such as rooftop silicon PV for the commercial and industrial scales.^14^ However, the following questions remain: *When considering the residential PV sector, what are the specific technology requirements for perovskite modules to be cost-competitive with c-Si modules, and are these specifications indeed less stringent than those considered for utility scale PV?How do perovskite–silicon (per-Si) tandem modules compare in this regard?* Finally, *which cost reductions can we take into consideration for the development of these new technologies into the future, for both perovskite single-junction (SJ) modules and per-Si tandem modules?*

To answer this set of questions, we investigate the potential for levelized cost of energy (LCOE) benefits in the residential solar market when moving from c-Si to perovskite or per-Si solar modules. This is illustrated in Figure 1. The residential market refers to PV systems with nominal power capacities below 10–30 kWp (equivalent to a surface of 50–150 m^2^ covered with 20% power conversion efficiency (PCE) solar panels), distinguishing it from utility-scale applications, where the power is above 1–10 MWp (equivalent to a 5,000–50,000 m^2^ surface of these same panels), and industrial-scale applications, which fall in between. To calculate the LCOE, we adopt the discounting method, which defines the LCOE as the ratio of the discounted costs to the discounted electricity generated throughout the entire lifespan of the rooftop PV system,^15^ as detailed in Section 1 of the Supporting Information (SI). This approach provides the advantage of explicitly taking into account the stability performance of the solar modules^16^—an essential metric for perovskite modules, as will become evident in the following sections. Consistent with our prior work, we divide the capital expenditures (CAPEX) into two segments, a module segment and a balance of system (BOS) segment, both paid in full in the initial year of installation.^17^

**Figure 1:**
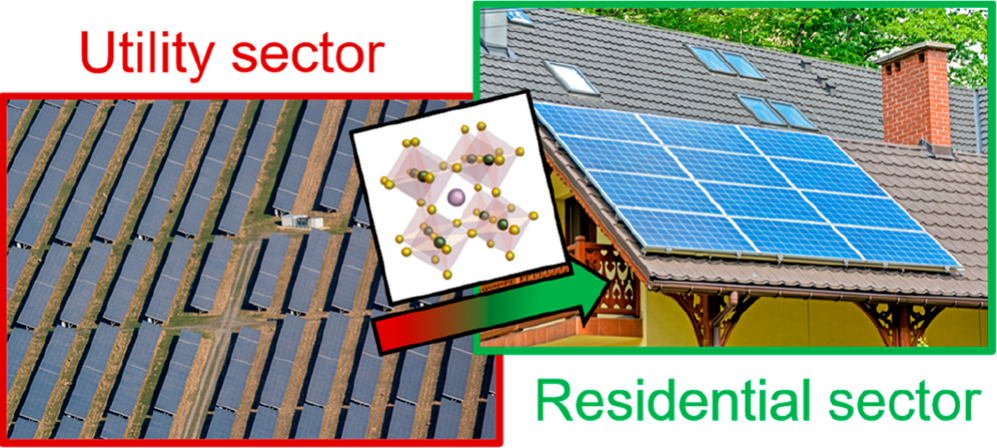
Will the residential sector be a less competitive market segment for perovskite photovoltaics than the utility sector?

For silicon PV, we set the total CAPEX at 1300 €_2021_/kWp,^18^ the operational expenditures (OPEX) at 26 €/kWp/yr,^18^ the residential PV system lifetime to 30 years,^18,19^ and the annual degradation rate (ADR) to 0.5%/yr.^18^ Of the total CAPEX, 40% is attributed to module costs, while the remaining 60% accounts for BOS costs.^20^ Under these conditions and for a solar irradiation of 1200 kWh/m^2^/yr, the LCOE of silicon PV for the residential sector is calculated at 11.7 ct/kWh. This value is higher than the 6.3 ct/kWh LCOE previously calculated for c-Si PV in the utility sector,^11^ due to higher CAPEX costs from BOS and higher OPEX, as well as a lower performance ratio of the modules. Specifically, factors contributing to the higher CAPEX and OPEX costs include economies-of-scale (where purchases in larger quantities lead to a lower cost per piece), labor costs (which are higher for case-by-case installations rather than for standardized installations), and soft costs (where individual assessments and permits are more complex than for streamlined processes).

The total CAPEX for perovskite PV is obtained by first determining the module contribution, which is calculated as the ratio of the modules’ cost over their efficiency. Specifically, we select three module cost scenarios—at 100, 50, and 25 €/m^2^—in order to represent the large variability in the yet-unknown final perovskite manufacturing cost.^11,21^ For the BOS contribution to CAPEX, we keep the same value as the one found for silicon PV (i.e., 780 €/kWp) but split this amount into a purely capacity-dependent cost and an efficiency-dependent cost; i.e., the former will stay fixed while the latter will decline with higher perovskite module PCE (see SI, Section 1, for more details). The resulting LCOE for the residential sector using perovskite modules is depicted in Figure 2 as a map, with the modules’ stability performance (ADR) swept from 0 to 10% on the *x*-axis and the modules’ efficiency performance (PCE) swept from 10 to 25% on the *y*-axis. For comparison purposes, the LCOE of c-Si modules in the residential sector is shown in red.

**Figure 2:**
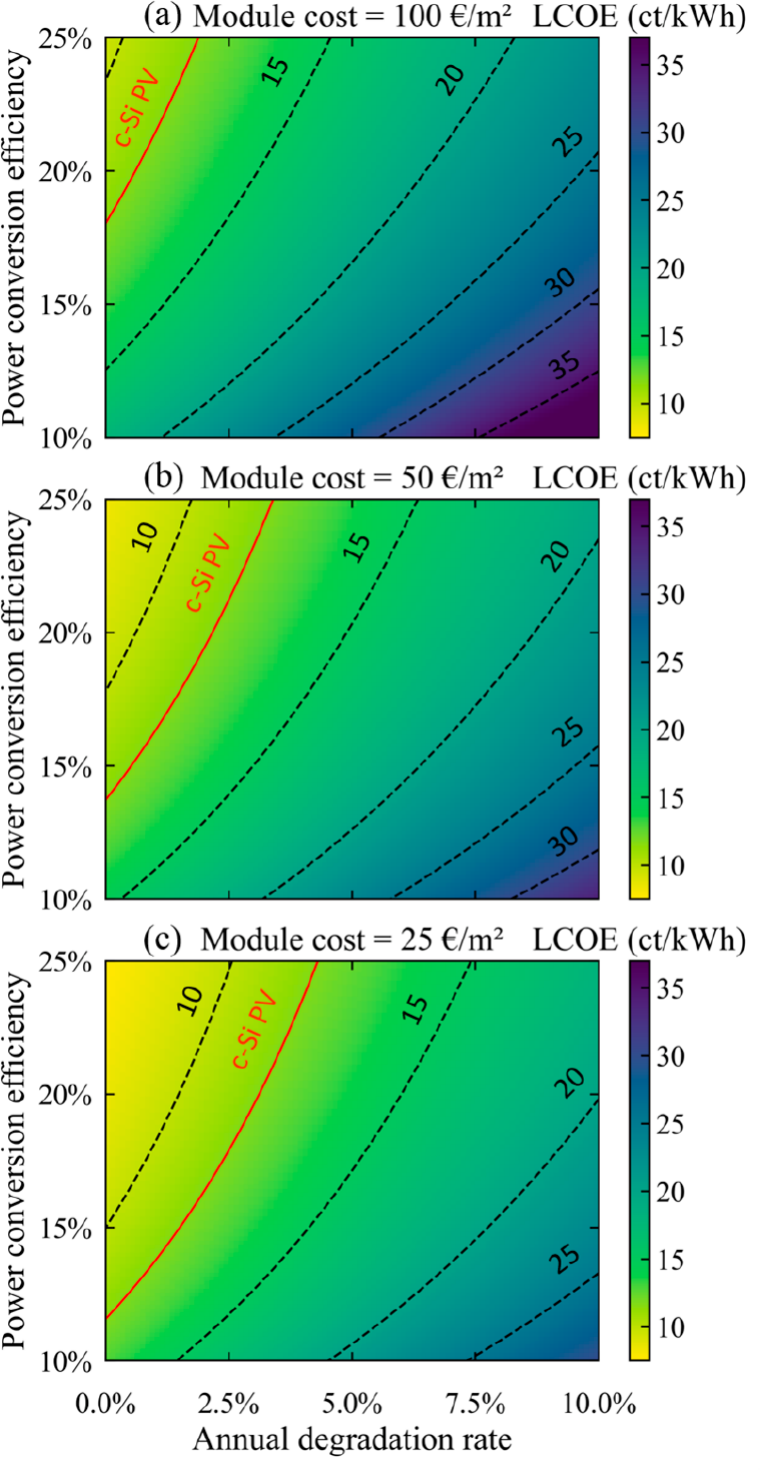
LCOE of single-junction perovskite modules, as a function of their PCE and ADR, for manufacturing costs of (a) 100, (b) 50, or (c) 25 €/m^2^.

We first notice the importance of the combination of cost, efficiency, and stability in allowing for an overall low LCOE and the large difference between the minimal LCOE obtainable, at 7.7 ct/kWh, and the maximal LCOE, at 40.7 ct/kWh. Compared to the utility sector, where perovskite modules costing 100 €/m^2^ were unable to compete with silicon modules,^11^ there is now a margin that enables their viability, when modules combine a PCE above 18% and an ADR below 2%. Similarly for perovskite modules at 50 (25) €/m^2^, which could previously only compete against silicon PV in the utility sector under conditions of a PCE above 18% (14.5%) and an ADR below 2.3% (3.4%), the constraints are now reduced to PCEs over 14% (11.5%) and ADRs below 3.5% (4.3%). In other words, if we consider a certain fixed cost for perovskite module production, the technical requirements for a net benefit against c-Si PV are lighter for the residential market than for the utility market. This substantially increases the potential for perovskite modules to enter the residential market compared to the utility market, although it does not necessarily guarantee a viable proposition on its own.

However, the most notable impact of the transition toward perovskite solar modules is not shown in this picture, and that is the increase in market potential for modules lighter than their silicon counterparts. Indeed, with roll-to-roll processing, perovskite modules can be deposited onto flexible substrates, typically made of plastic polymers, resulting in much lighter modules than the majority of existing silicon alternatives^22,23^ (see SI, Section 1). This enables installation of PV panels on rooftops that previously could not support the weight of traditional panels, making cost competition against silicon PV irrelevant in these cases. Light-weight perovskite modules might thus mark the initial phase of perovskite market growth, specifically in the context of buildings with low structural integrity. This could potentially pave the way for broader perovskite adoption within the residential sector, provided that single-junction modules achieve the desired combination of high PCE and low ADR as examined above, to effectively offer a net LCOE benefit over silicon modules.

Perovskite SJ modules are only one of the applications of perovskite materials for solar PV. Another promising avenue of research for perovskite materials lies in their integration together with silicon to form per-Si tandem modules.^24^ Despite what these new tandem modules might lose in the light weight and flexibility of the SJ modules, they offer the advantage of increasing the theoretical PCE above the detailed balance limit^25,26^—with a current record of 33.7%^27^—and, when combined with a silicon sub-cell, they can benefit from leveraging a mature and well-established technology. To evaluate the LCOE benefits of per-Si tandem modules compared to conventional c-Si modules in the residential sector, we calculate the LCOE as a function of the modules’ potential stability and efficiency performances. The LCOE mapping methodology proposed earlier is slightly modified: instead of the 10–25% PCE sweep used for the SJ perovskite modules, the per-Si tandem module PCE is now swept from 20% to a maximal 40%, and the overall module cost is increased by a fixed 50 €/m^2^ to account for the additional silicon sub-cell cost (see SI, Section 1). The remaining components of the analysis, including the BOS and OPEX costs, are kept unchanged. Figure 3 illustrates these LCOE maps for per-Si tandem modules in the residential sector, considering module cost scenarios of 150, 100, and 75 €/m^2^. The LCOE of c-Si modules is highlighted in red.

**Figure 3:**
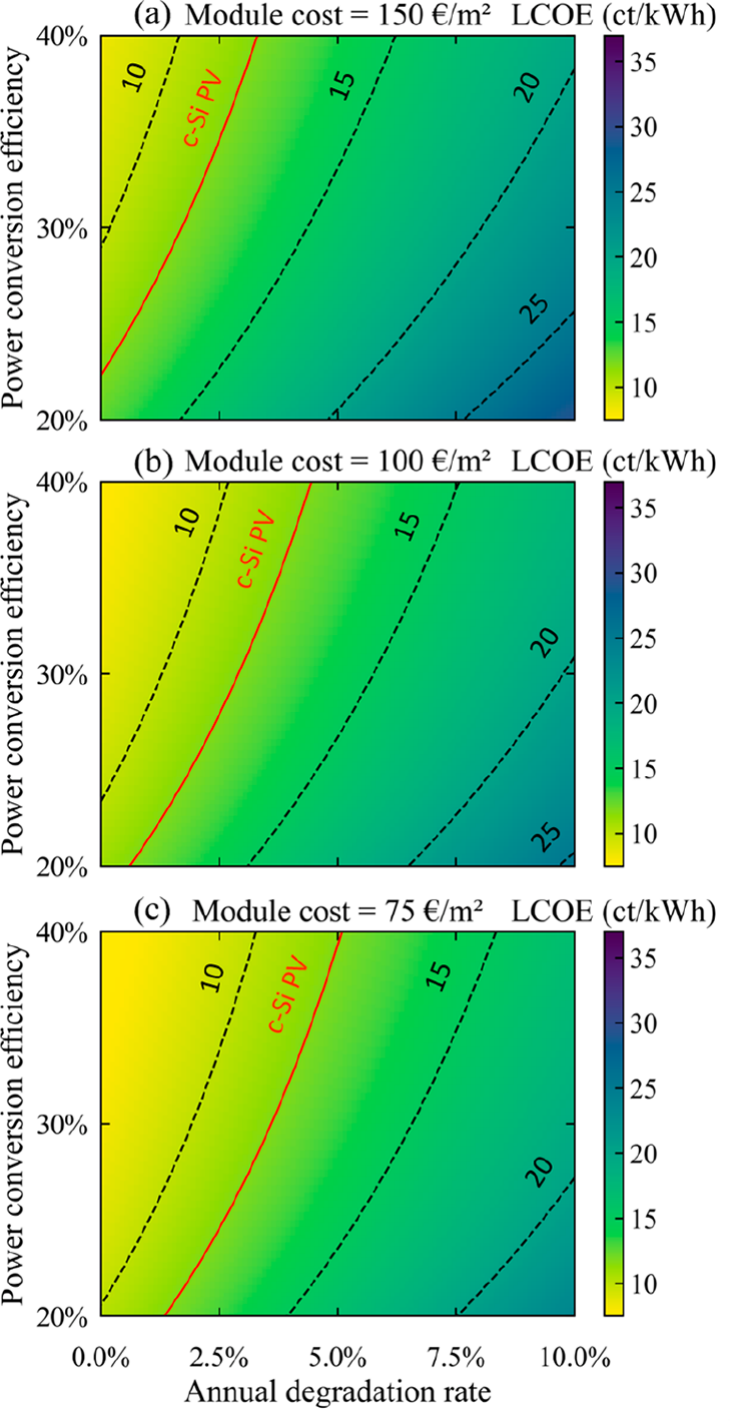
LCOE of per-Si tandem modules, as a function of their PCE and ADR, for manufacturing costs of (a) 150, (b) 100, or (c) 75 €/m^2^.

Again, achieving an advantageous LCOE requires a combination of high efficiency, high stability, and low cost. In this case, the minimum LCOE achieved is 7.3 ct/kWh, which is comparable to the minimum LCOE obtained for SJ perovskite modules (7.7 ct/kWh). However, the conditions required to reach low LCOEs, especially those competitive with the 11.7 ct/kWh threshold of c-Si PV, are less stringent than in the case of SJ perovskite modules. Indeed, the maximum ADRs are increased to 3.3, 4.5, and 5% (compared to 2, 3.5, and 4.3%) in the respective three module cost scenarios. This phenomenon is also visible in the maximum LCOE achieved, which is lower, here at 29 ct/kWh. In other words, the increase in module cost for tandems is offset by the possibility of reaching a higher PCE with these modules. Overall, in the residential sector, any per-Si tandem module with a module cost equal to or below 100 €/m^2^ and an ADR below 1% would be competitive with c-Si PV, provided its PCE exceeds 20%. Compared to the utility sector, where competition against c-Si PV could only be achieved for PCEs above 35, 26.5, and 22.5% and ADRs below 1, 2.6, and 3.7%, respectively for tandem modules costing 150, 100, and 75 €/m^2^, here competition can happen when the PCE is above 22.5% or 20% and when the ADR is below 3.3, 4.5, or 5%. The conditions for competition against c-Si PV are thus significantly relaxed when compared with those in the utility sector, in terms of both efficiency and stability.

Per-Si tandem modules can thus be competitive with c-Si PV across a broader range of stability performances than the SJ perovskite modules, thanks to their higher efficiency metric. On the other hand, perovskite SJ modules have the potential to explore markets previously untapped for c-Si PV, thanks to their light weight and flexibility, allowing for installation on a wider variety of rooftops. In the long run, perovskite/perovskite tandem modules could combine the benefits of both systems.

Finally, we look into potential cost reductions in the residential sector for both SJ perovskite and per-Si tandem modules. We use the learning curve methodology^28^ in conjunction with an anticipated increase in PCE over time.^11,29^ We begin our analysis from the year 2025, considering an initial cumulative installed capacity of 1 GWp. In the baseline scenario, the learning rates are set to 25%^30^ for module costs and to 10% for BOS costs,^31^ while the compound annual growth rate (CAGR) is set to 25%.^32,33^ The optimistic scenario assumes a learning rate of 30% for modules and 15% for BOS, and a CAGR of 30%, while the conservative scenario assumes a learning rate of 20% for modules and 5% for BOS, and a CAGR of 20%. For the initial module cost in 2025, we consider three distinct values: the medium and high values indicated in the module cost scenarios presented in Figures 2 and 3, along with the average value derived from both scenarios. The remaining assumptions in terms of BOS costs and OPEX are elaborated upon in Section 2 of the SI (see Tables S1 and S2), together with the cost reductions in terms of module CAPEX and BOS CAPEX (see Figures S1 and S2).

For perovskite SJ modules, the initial PCE is set at 12.5, 15, or 17.5%, respectively in the conservative, baseline, and optimistic scenarios, and grows with an annual progress rate of 0.2, 0.3, or 0.4%/yr. Figure 4 shows the LCOE reduction for these modules: from 8.6, 15, and 24 ct/kWh in 2025 to 4.6, 8.7, and 15.9 ct/kWh in 2050. For comparison purposes, we estimate the LCOE of c-Si PV in 2050, and find 7.3 ct/kWh (see SI, Section 2). Thus, not only do perovskite SJ modules offer the possibility of creating new markets for rooftop residential PV, but on top of that, these new markets might additionally be cost-competitive with traditional c-Si PV, at least under the optimistic scenario conditions presented here.

**Figure 4:**
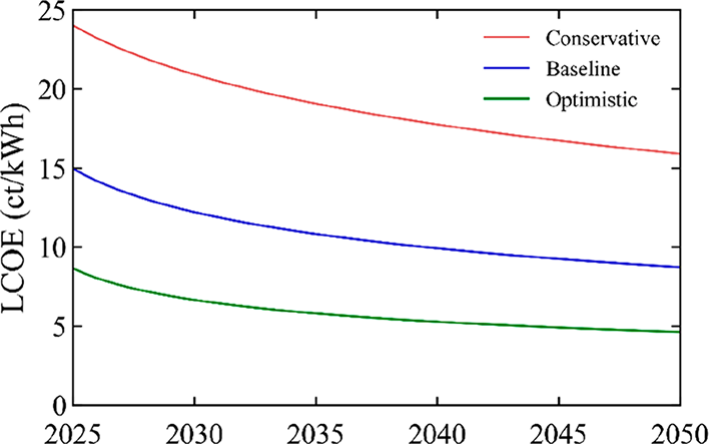
LCOE of SJ perovskite modules in the residential market under conservative, baseline, and optimistic scenarios, for the time period 2025–2050. The LCOE is calculated for an average irradiation of 1200 kWh/m^2^/yr.

For per-Si tandem modules, the initial PCE is set at 20, 22.5, or 25%, respectively in the conservative, baseline, and optimistic scenarios. Figure 5 illustrates the LCOE cost reduction scenarios for these modules. Notably, we observe a significant decrease from 8.4, 13.5, and 20 ct/kWh in 2025 to 4.6, 8.3, and 14.3 ct/kWh in 2050. These findings reaffirm the potential of per-Si tandem modules to achieve lower LCOEs compared to their SJ perovskite module counterparts, as visible in the baseline and conservative frameworks. However, it is worth noting that both technologies exhibit the same minimal achievable LCOE in the optimistic frameworks, highlighting the value of both options for future applications in the residential PV market.

**Figure 5:**
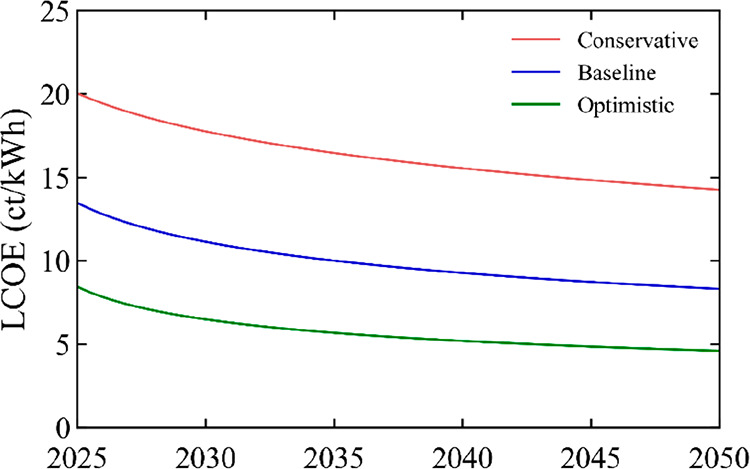
LCOE of per-Si tandem modules in the residential market under conservative, baseline, and optimistic scenarios, for the time period 2025–2050. The LCOE is calculated for an average irradiation of 1200 kWh/m^2^/yr.

In conclusion, we argue that flexible perovskite SJ modules offer unique advantages by expanding into market segments that were previously inaccessible to c-Si PV. These segments include rooftops with low structural integrity as well as those with specific tilting and complex geometries. In such cases, the light weight and flexibility of these new perovskite SJ modules add significant value to the PV market, making them desirable products beyond their potential for low LCOE. They do, additionally, still hold the potential for competition against c-Si PV, but only under a specific intersection of low cost, high stability, and high efficiency. Per-Si tandems, on the other hand, can achieve lower LCOEs than c-Si PV under a wider set of performance requirements than their SJ counterparts, especially considering stability. Overall, we find that the technology requirements for perovskite-containing modules are relaxed in the residential sector compared to those in the utility sector. The larger LCOE of c-Si PV in this sector is thus offset by the lower module costs that can be achieved with perovskite materials.

As we envision the future of solar PV, our learning curve analysis shows that there is considerable potential for cost reductions in perovskite SJ and per-Si tandem modules, achieved by both improving module efficiency and reducing CAPEX. Under our optimistic scenario, the LCOEs can reach as low as 4.6 ct/kWh by 2050. Compared to the modeled LCOE of 7.3 ct/kWh for c-Si PV in the residential sector in 2050, both perovskite technologies would thus have the ability to compete against this established technology.

## Supporting Information Available

Methods and data sets for the LCOE calculation; cost reduction scenarios analysis (PDF)
