# Supplementary material for: Perovskite Solar Modules for the Residential Sector
Source: ACS Energy Lett. 2023 Oct 25;8(11):4862–6. doi: 10.1021/acsenergylett.3c02111 (PMC10644372; doi:10.1021/acsenergylett.3c02111)
Supplement: Supplementary file 1 — nz3c02111_si_001.pdf [file nz3c02111_si_001.pdf]

Supporting Information to

Perovskite Solar Modules for the Residential Sector

Lucie McGovern[1,2,*], Esther Alarcón-Lladó[1,2], Erik C. Garnett[1,2], Bruno Ehrler[2] and Bob van der Zwaan[1,3,4]

[1] Faculty of Science (HIMS, IOP and/or IAS), University of Amsterdam, Amsterdam, 1098 XH, The Netherlands

[2] Center for Nanophotonics, AMOLF, Amsterdam, 1098 XG, The Netherlands

[3] TNO Energy Transition Studies, Amsterdam, 1043 NT, The Netherlands

[4] School of Advanced International Studies (SAIS), Johns Hopkins University, Bologna, 40126, Italy

[*] Corresponding author: lucie.mcgovern@free.fr

Section 1. Methods and datasets for the LCOE calculation

In Figures 2 and 3, we calculate the LCOE as a function of solar modules' PCE and ADR. The full equation is presented below:

$$\mathrm{LCOE} = \frac{\mathrm{CAPEX} + \sum_{t=1}^{T} \frac{\mathrm{OPEX}}{(1+\delta)^{t-1}}}{\sum_{t=1}^{T} \frac{\mathrm{PR} \times \mathrm{Irr} \times (1-\mathrm{ADR})^{t-1}}{(1+\delta)^{t-1}}},$$

where CAPEX (in $€_{2021}$/kWp) are the capital expenditures incurred for the installation of the PV residential solar system at time $t = 0$, OPEX (in $€_{2021}$/kWp/yr) are the yearly operational expenditures, δ (in %) is the discount rate, PR (in %) is the performance ratio of the PV modules, Irr (in kWh/m^2/yr) is the local irradiance, ADR (in %) is the annual degradation rate of the PV modules, and T (in yr) is the total lifetime of the project.

As mentioned in the main text, for our study we use total CAPEX of 1300 $€_{2021}$/kWp[1] for c-Si PV. This value is the average of the high-end value (1600 $€_{2021}$/kWp) and low-end value (1000 $€_{2021}$/kWp) reported in the Fraunhofer study of 2021[1]. Additionally, we consider $\delta = 5\%$[2] and $\mathrm{PR} = 80\%$. Compared to the 85% PR used in our work on the utility sector[3], the lower PR chosen here represents the higher losses occurring from shading and soiling in urban area contexts, relative to the more ideal conditions set forward in the utility context.

When calculating the BOS costs for modules containing perovskite materials, we use the baseline of 780 €/kWp reported for c-Si PV in the residential sector[1]. Of these 780 €/kWp, 70% (i.e. 546 €/kWp) are attributed to BOS costs dependent on the modules' efficiency while the remaining 30% (i.e. 234 €/kWp) are attributed to BOS costs dependent on the PV system power output[4]. The efficiency-dependent segment will reduce with higher perovskite PCE, i.e. $\mathrm{BOS} = 546 \times \frac{\mathrm{PCE\ (silicon)}}{\mathrm{PCE\ (perovskite)}} + 234.$ We consider an average c-Si PCE of 20% for the residential sector.

For the per-Si tandem cost calculation, we select an additional cost of 50 €/m^2 for the silicon sub-cell, corresponding to the minimal price found online for purchasing a single silicon solar module[5], and equivalent to the manufacturing cost of silicon sub-cells in the utility scale[3]. For simplicity purposes, we don't consider any additional cost for the integration of the two sub-cells. We note, however, that the balance of modules components (junction box, wiring, etc) are accounted for twice in our calculation (in the perovskite module cost, as well as in the additional silicon sub-cell cost). This effect is expected to counterbalance the initial simplified assumption.

In the main text, we report on perovskite solar modules being the key lightweight alternative to the majority of existing c-Si technologies. We do, however, also acknowledge the recent emergence of lightweight c-Si PV modules in the market, such as those employing metal wrap through designs[6] together with lighter glass covers and frame. So far, these modules only represent a very small fraction of the c-Si PV modules on the market, but we cannot exclude the possibility of this market share growing in future years.

Section 2. Cost reduction scenarios analysis

In Tables S1 and S2 below, we present the assumptions used to derive the different cost reduction scenarios shown in Figure 4 and 5 of the main text, respectively for perovskite SJ and per-Si tandem modules.

The initial BOS CAPEX are set to 40, 60, and 80% of the total CAPEX for c-Si PV in the optimistic, baseline and conservative scenarios, including a contribution of either 32, 42, or 52% which is efficiency-dependent, and a remaining contribution of 8, 18 or 28% which is purely capacity-dependent. The ADR of the modules are set to 1%, 2% and 3%, respectively.

	Conservative	Baseline	Optimistic
LR CAPEX module [%]	20	25	30
LR CAPEX BOS [%]	5	10	15
PCE module [%]	12,5	15	17,5
APR PCE module [%/yr]	0,2	0,3	0,4
CAGR [%]	20	25	30
Initial CIC [GW]	1	1	1
Initial CAPEX module [€/m^2]	100	75	50
Initial CAPEX BOS (c) [€/kW]	135,2	109,2	83,2
Initial CAPEX BOS (a) [€/m^2]	364	234	104
OPEX [€/kW/yr]	31	26	21
ADR [%/yr]	3	2	1

Table S1. Set of assumptions regarding module CAPEX learning rate, BOS CAPEX learning rate, module PCE, module PCE annual progress rate (APR), CAGR, initial cumulative installed capacity (CIC), initial module CAPEX, initial BOS CAPEX (capacity-dependent and area-dependent), and ADR for the conservative, baseline and optimistic cost reduction scenarios of SJ perovskite modules, as shown in Figure 4.

	Conservative	Baseline	Optimistic
LR CAPEX module [%]	20	25	30
LR CAPEX BOS [%]	5	10	15
PCE module [%]	20	22,5	25
APR PCE module [%/yr]	0,2	0,3	0,4
CAGR [%]	20	25	30
Initial CIC [GW]	1	1	1
Initial CAPEX module [€/m^2]	150	125	100
Initial CAPEX BOS (c) [€/kW]	135,2	109,2	83,2
Initial CAPEX BOS (a) [€/m^2]	364	234	104
OPEX [€/kW/yr]	31	26	21
ADR [%/yr]	3	2	1

Table S2. Set of assumptions regarding module CAPEX learning rate, BOS CAPEX learning rate, module PCE, module PCE annual progress rate (APR), CAGR, initial cumulative installed capacity (CIC), initial module CAPEX, initial BOS CAPEX (capacity-dependent and area-dependent), and ADR for the conservative, baseline and optimistic cost reduction scenarios of per-Si tandem modules, as shown in Figure 5.

In Figure S1, we show the cost reductions for SJ perovskite modules from 2025 to 2050, specifically in terms of the module CAPEX and BOS CAPEX, according to the three scenarios described above. From 2025 to 2050, module CAPEX reduce from 800 to 320 €/kWp in the conservative scenario, from 333 to 98 €/kWp in the baseline scenario, and from 142 to 30 €/kWp in the optimistic scenario. For BOS CAPEX, the costs are reduced from 1445 to 995 €/kWp in the conservative scenario, from 962 to 532 €/kWp in the baseline scenario, and from 580 to 246 €/kWp in the optimistic scenario. The module CAPEX thus reduces much faster than the BOS CAPEX, a phenomenon which can be explained by the lower learning rate applied to BOS compared to modules.

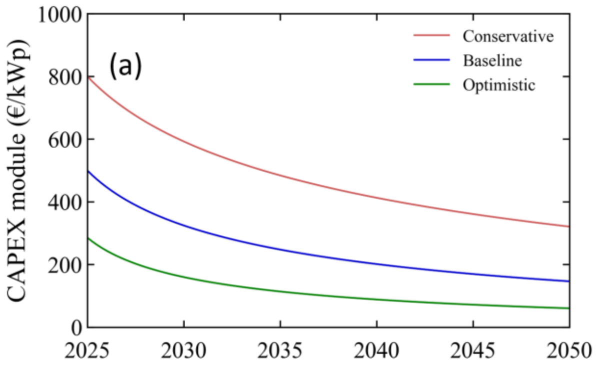

Figure S1. Cost reductions for SJ perovskite modules in the residential market, under conservative, baseline, and optimistic scenarios, for the time period 2025 – 2050, in terms of (a) the module CAPEX and (b) the BOS CAPEX.

In Figure S2, we show the cost reductions in terms of module CAPEX and BOS CAPEX for per-Si tandem modules, according to the same three scenarios, from conservative to optimistic. From 2025 to 2050, module CAPEX reduce from 750 to 337 €/kWp in the conservative scenario, from 444 to 147 €/kWp in the baseline scenario, and from 142 to 30 €/kWp in the optimistic scenario. For BOS CAPEX, the costs are reduced from 1040 to 792 €/kWp in the conservative scenario, from 720 to 442 €/kWp in the baseline scenario, and from 300 to 71 €/kWp in the optimistic scenario. The BOS CAPEX are lower than in the case of SJ perovskite modules, due to the higher PCEs reached by the tandem modules.

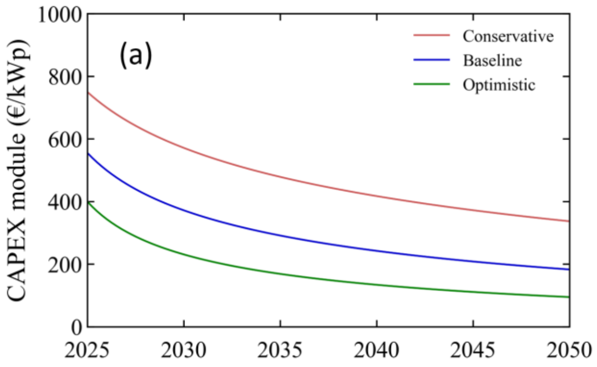

Figure S2. Cost reductions for per-Si tandem modules in the residential market, under conservative, baseline, and optimistic scenarios, for the time period 2025 – 2050, in terms of (a) the module CAPEX and (b) the BOS CAPEX.

In order to compare the LCOE of SJ perovskite modules and per-Si tandem modules to that of c-Si PV modules in 2050 in the residential sector, we calculate the LCOE of c-Si PV for 2050. We consider the PCE of c-Si PV to grow by a 0.2%/yr rate, which, with the initial PCE set at 20% in 2025, is equivalent to having modules with 25% PCE by 2050. Over this same time period, the ADR of c-Si PV is decreased from 0.05%/yr to 0.025%/yr - a value already common for c-Si modules in the industrial and utility sectors. The LRs for module and BOS are kept equal to those from the baseline scenarios of perovskite-containing modules, i.e. 25% and 10%. We obtain a 7.3 ct/kWh LCOE for c-Si PV in 2050.

References

(1) Fraunhofer ISE. *Levelized Cost Of Electricity. Renewable Energy Technologies.*; 2021.

(2) Renewable Power Generation Costs in 2021 https://www.irena.org/publications/2022/Jul/Renewable-Power-Generation-Costs-in-2021 (accessed Nov 22, 2022).

(3) McGovern, L.; Garnett, E. C.; Veenstra, S.; Zwaan, B. van der. A Techno-Economic Perspective on Rigid and Flexible Perovskite Solar Modules. *Sustain. Energy Fuels* **2023**. https://doi.org/10.1039/D3SE00828B.

(4) Zafoschnig, L. A.; Nold, S.; Goldschmidt, J. C. The Race for Lowest Costs of Electricity Production: Techno-Economic Analysis of Silicon, Perovskite and Tandem Solar Cells. *IEEE J. Photovoltaics* **2020**, *10* (6), 1632–1641. https://doi.org/10.1109/JPHOTOV.2020.3024739.

(5) 25 Years Warranty Poly 300w https://www.alibaba.com/product-detail/The-Solar-Panel-25-Years-Warranty_60800531879.html?spm=a2700.galleryofferlist.0.0.35a55507zMqX54 (accessed Jul 21, 2023).

(6) Guillevin, N.; Heurtault, B. J. B.; Van Aken, B. B.; Bennett, I. J.; Jansen, M. J.; Berkeveld, L.; Geerligs, L. J.; Weeber, A. W.; Bultman, J. H.; Hu, Z.; Li, G.; Zhao, W.; Wang, J.; Wang, Z.; Chen, Y.; Shen, Y.; Chen, J.; Yu, B.; Tian, S.; Xiong, J. High Efficiency N-Type Metal Wrap through Cells and Modules. *Energy Procedia* **2012**, *27*, 610–616. https://doi.org/10.1016/J.EGYPRO.2012.07.118.
